# Supplementary material for: Cobalt Phthalocyanine-Doped Polymer-Based Electrocatalyst for Rechargeable Zinc-Air Batteries
Source: Materials (Basel). 2023 Jul 20;16(14):5105. doi: 10.3390/ma16145105 (PMC10386096; doi:10.3390/ma16145105)
Supplement: Supplementary file 1 [file materials-16-05105-s001.zip › materials-2509104-supplementary.pdf]

materials

Supplementary Materials

Cobalt Phthalocyanine-Doped Polymer-Based Electrocatalyst for Rechargeable Zinc-Air Batteries

Yogesh Kumar [1], Srinu Akula [1], Elo Kibena-Põldsepp [1], Maike Käärik [1], Jekaterina Kozlova [2], Arvo Kikas [2], Jaan Aruväli [3], Vambola Kisand [2], Jaan Leis [1], Aile Tamm [2] and Kaido Tammeveski [1,*]

[1] Institute of Chemistry, University of Tartu, 50411 Tartu, Estonia; yogesh.kumar@ut.ee (Y.K.); srinu.akula@ut.ee (S.A.); elo.kibena-poldsepp@ut.ee (E.K.-P.); maike.kaarik@ut.ee (M.K.); jaan.leis@ut.ee (J.L.)
[2] Institute of Physics, University of Tartu, 50411 Tartu, Estonia; jekaterina.kozlova@ut.ee (J.K.); arvo.kikas@ut.ee (A.K.); vambola.kisand@ut.ee (V.K.); aile.tamm@ut.ee (A.T.)
[3] Institute of Ecology and Earth Science, University of Tartu, 50409 Tartu, Estonia; jaan.aruvali@ut.ee
* Correspondence: kaido.tammeveski@ut.ee

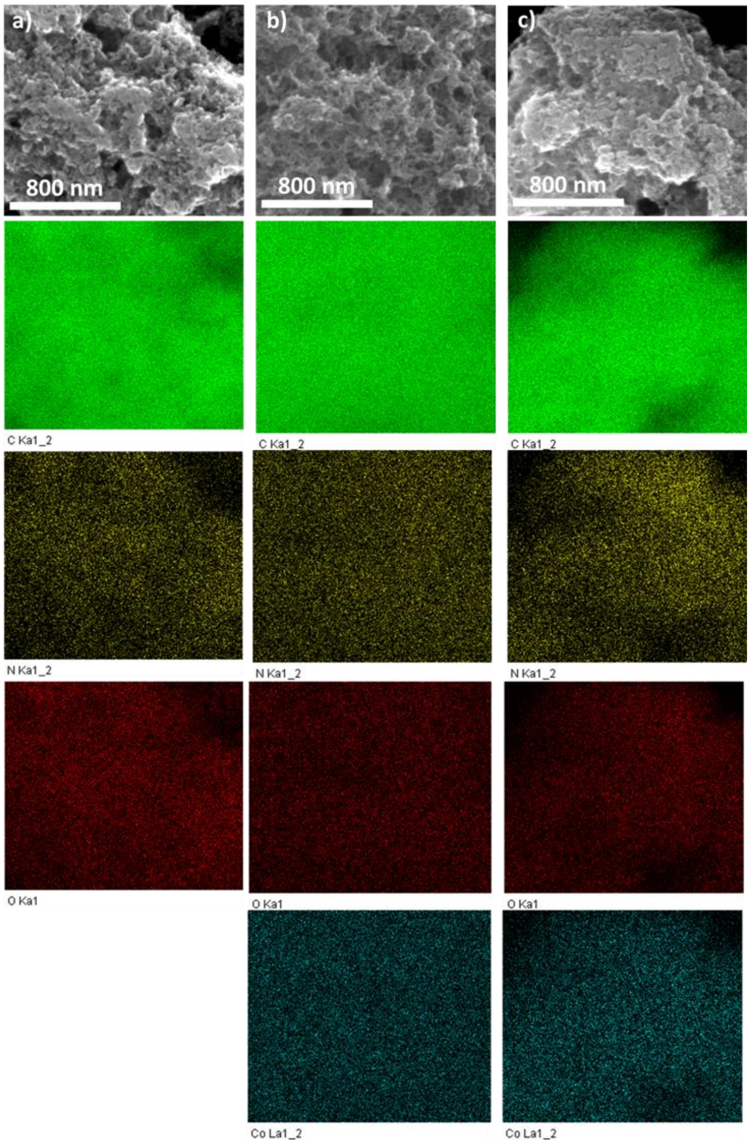

Figure S1. Elemental maps of C, N, O, and Co obtained from SEM-EDX of (a) PC-800, (b) CoN-PC-1, and (c) CoN-PC-2.

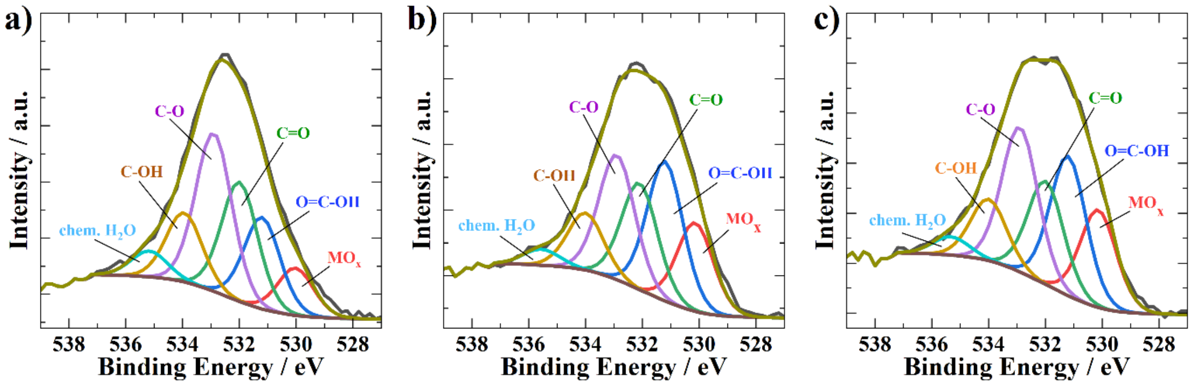

Figure S2. O 1s high-resolution XPS spectra of a) PC-800, b) CoN-PC-1, and c) CoN-PC-2.

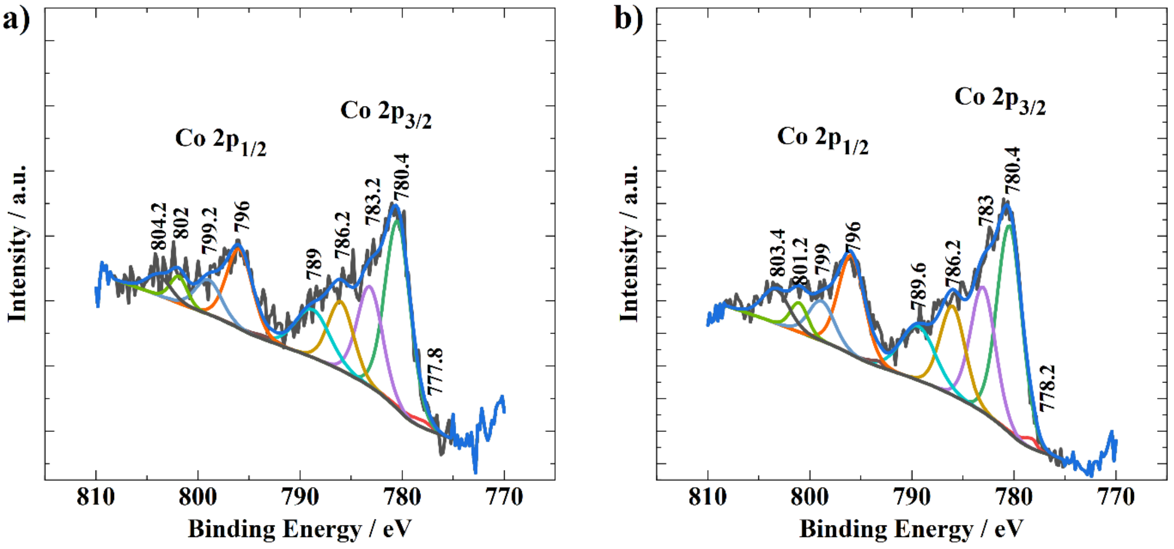

Figure S3. Deconvoluted Co 2p XPS spectra for a) CoN-PC-1 and b) CoN-PC-2 catalysts.

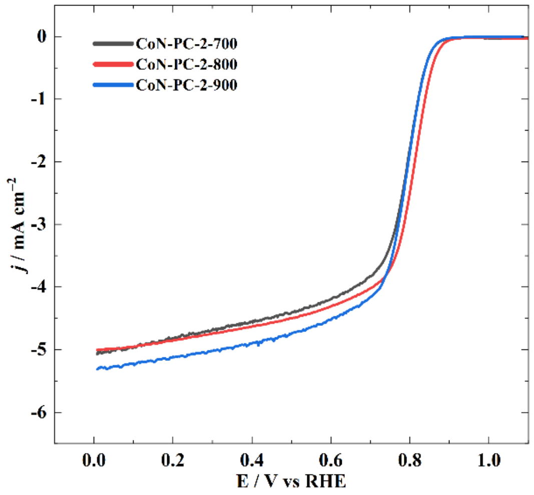

Figure S4. ORR polarisation curves for catalysts prepared at different temperatures. Test conditions: O_2-saturated 0.1 M KOH solution, ν = 10 mV s^{-1}, ω = 1900 rpm.

Table S1. ORR parameters in 0.1 M KOH for catalysts prepared at different temperatures.

Catalyst	E_{onset} / V	$E_{1/2}$ / V
**CoN-PC-2-700**	0.87	0.78
**CoN-PC-2-800**	0.88	0.81
**CoN-PC-2-900**	0.88	0.78

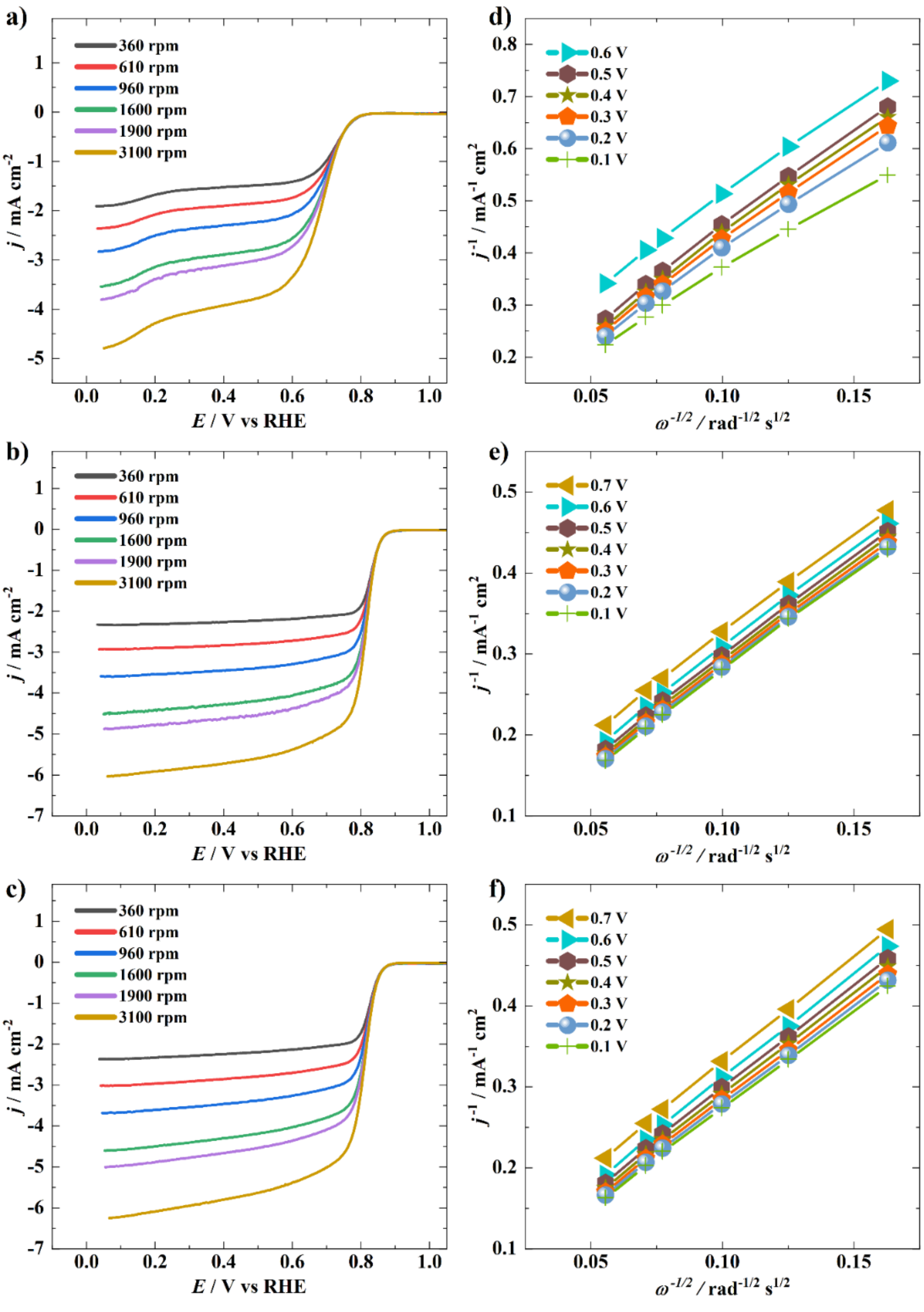

Figure S5. ORR polarisation curves at different rotation rates for (a) PC-800, (b) CoN-PC-1, and (c) CoN-PC-2 in O_2-saturated 0.1 M KOH solution. Koutechy–Levich (K-L) plots for (d) PC-800, (e) CoN-PC-1, and (f) CoN-PC-2 derived from the respective polarisation curves.

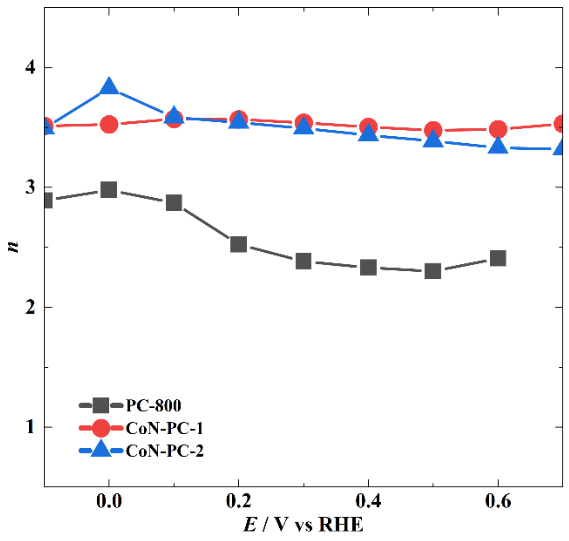

Figure S6. Number of transferred electrons per O_2 molecule derived from the K-L plots.

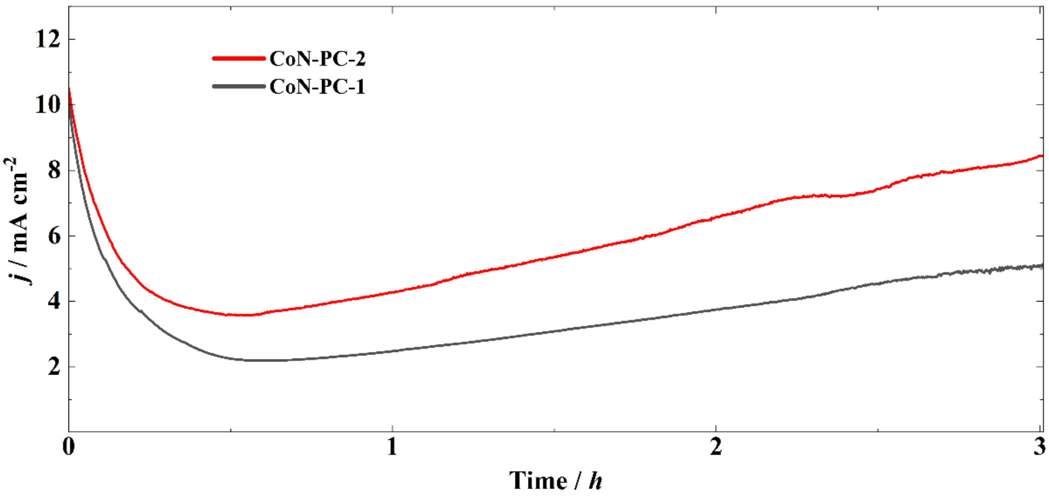

Figure S7. OER stability test for CoN-PC-1 and CoN-PC-2 at 1.7 V for 3 h (ω = 1900 rpm).
